# Divergent Evolution of Volatile Compounds in Wild Ginseng Across Growth Years: Terpene Accumulation and Overall Pyrazine Decline Revealed by HS-GC-IMS

**DOI:** 10.3390/molecules31132315

**Published:** 2026-07-01

**Authors:** Lili Cui, Hongying Guo, Yuhe Ren, Rui Wang, Meiling Jin, Tianxing Zhao, Ze Zhang, Xuan Li, Hui Zhao

**Affiliations:** Institute of Special Wild Economic Animal and Plant Science, Chinese Academy of Agricultural Sciences, Changchun 130112, China; cbscui@126.com (L.C.); gerhyy@163.com (H.G.); renyuhe1123@163.com (Y.R.); 18744108018@163.com (R.W.); lovemeiling521@hotmail.com (M.J.); zhaotx_1998@163.com (T.Z.); zz51998@126.com (Z.Z.); ndlixuan@163.com (X.L.)

**Keywords:** wild ginseng, growth years, quality assessment, flavor compounds, gas chromatography-ion mobility spectrometry (GC-IMS), chemical fingerprint, partial least squares-discriminant analysis (PLS-DA)

## Abstract

The volatile compounds (VOCs) evolution of wild ginseng (WG) across growth years is not a unidirectional process but a divergent remodeling of the chemical fingerprint. In this study, HS-GC-IMS combined with chemometrics was employed to characterize the dynamic changes in VOCs in WG at four growth stages (10, 15, 20, and 25 years). Samples were collected from three independent sites, with ≥5 roots pooled per site per age group to obtain population-representative composite samples. A total of 68 VOCs were tentatively identified and semi-quantified. Terpenes and pyrazines exhibited the most pronounced directional trends: terpenes such as camphene, (E,E)-α-farnesene, and β-ionone accumulated progressively (increases of 242%, 74.6%, and 93.4%, respectively), whereas pyrazines showed an overall decline through distinct trajectories—2,3,5-trimethylpyrazine declined monotonically (57.3%), 2-ethyl-6-methylpyrazine decreased moderately (27.7%), and 2,5-dimethylpyrazine exhibited a transient peak at 20 years before a sharp terminal decline (52.3%). In contrast, the majority of compounds (66%) displayed non-monotonic patterns, underscoring the complexity of this divergent evolution. Partial least squares-discriminant analysis (PLS-DA) effectively distinguished samples across growth years (R^2^Y = 0.997, Q^2^ = 0.993), with a 200-times permutation test confirming no overfitting. 29 differential compounds (VIP > 1) were identified. The divergent accumulation patterns of terpenes and pyrazines, together with 29 VIP-identified differential compounds, provide chemical evidence that the VOCs fingerprint could serve as a supplementary tool for growth-year assessment. These findings provide chemical evidence that WG flavor quality evolves divergently over time, suggesting that VOCs fingerprint could serve as a supplementary tool for growth-year assessment, particularly for “high-quality but poor-shape” specimens that are undervalued by traditional morphology-based methods.

## 1. Introduction

Wild ginseng (WG, *Panax ginseng* C. A. Mey.) is a slow-growing perennial herb native to Northeast Asia. In 2018, global ginseng production was about 86,223 tons based on fresh ginseng; the value of global ginseng production is estimated to be approximately $5900 million [1]. Ginseng trade is projected to reach approximately $17.7 billion by 2030 [2]. Its volatile organic compounds (VOCs)—particularly terpenes, pyrazines, and aldehydes—largely determine the product’s aromatic quality and contribute to reported health benefits, including antioxidant and anti-inflammatory activities [3].

Current commercial grading of WG relies almost exclusively on morphological traits (the “five shapes”) to infer growth years, which are the core pricing criterion [4,5,6]. This system has two critical shortcomings. First, it systematically undervalues “high-quality but poor-shape” roots—specimens that possess sufficient growth years and abundant secondary metabolites yet fail to meet conventional morphological standards. Our field surveys and discussions with local growers consistently indicate that such roots represent a substantial proportion of harvests, making this a commercially significant problem rather than a marginal occurrence. Second, it impedes the market for processed WG products (e.g., powder) because comminution destroys morphological features and leaves consumers without objective criteria to verify authenticity and age. An alternative approach based on chemical profiling—applicable to powder regardless of root appearance—would address both problems. VOCs, which integrate the effects of age and environmental stress [7], represent a promising chemical fingerprint for this purpose.

Previous studies have shown that ginsenoside content increases with growth years in WG [8,9], and transcriptomic data suggest age-related upregulation of terpene biosynthesis genes. Phytosterol content in forest-cultivated ginseng—which shares the same species identity as WG—also correlates positively with growth years [10], and given the conserved isoprenoid pathways, a similar accumulation of terpenoid VOCs in WG is plausible but unproven. However, direct evidence on long-term VOC evolution in authentic WG (10–25 years) is scarce. Existing data are limited to short-term studies on cultivated ginseng [10] and cannot be extrapolated to decades-old WG. No study has systematically characterized whether different VOC classes follow divergent age-related trajectories, nor has a chemical fingerprint-based model been proposed for growth-year discrimination of WG powder.

To fill these gaps, we profiled VOCs from WG powder across four growth-year cohorts (10, 15, 20, and 25 years) using HS-GC-IMS combined with chemometrics. We hypothesize that: (i) terpenes will increase monotonically with age, driven by sustained MVA/MEP pathway activity; (ii) pyrazines will show an overall decrease from 10 to 25 years, though the temporal trajectories may differ among individual compounds—with some declining monotonically and others exhibiting non-monotonic fluctuations—reflecting a long-term attenuation of nitrogen metabolism with possible stage-specific metabolic events; and (iii) the majority of remaining VOCs will exhibit non-monotonic fluctuations. The objectives were to: (1) characterize VOC dynamics across the four age groups; (2) identify differential markers via PLS-DA.

## 2. Results

### 2.1. Flavor Compounds Identification and Spectral Features

**Figure 1:**
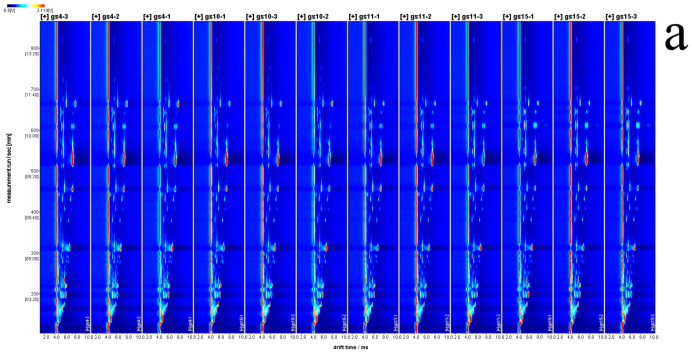

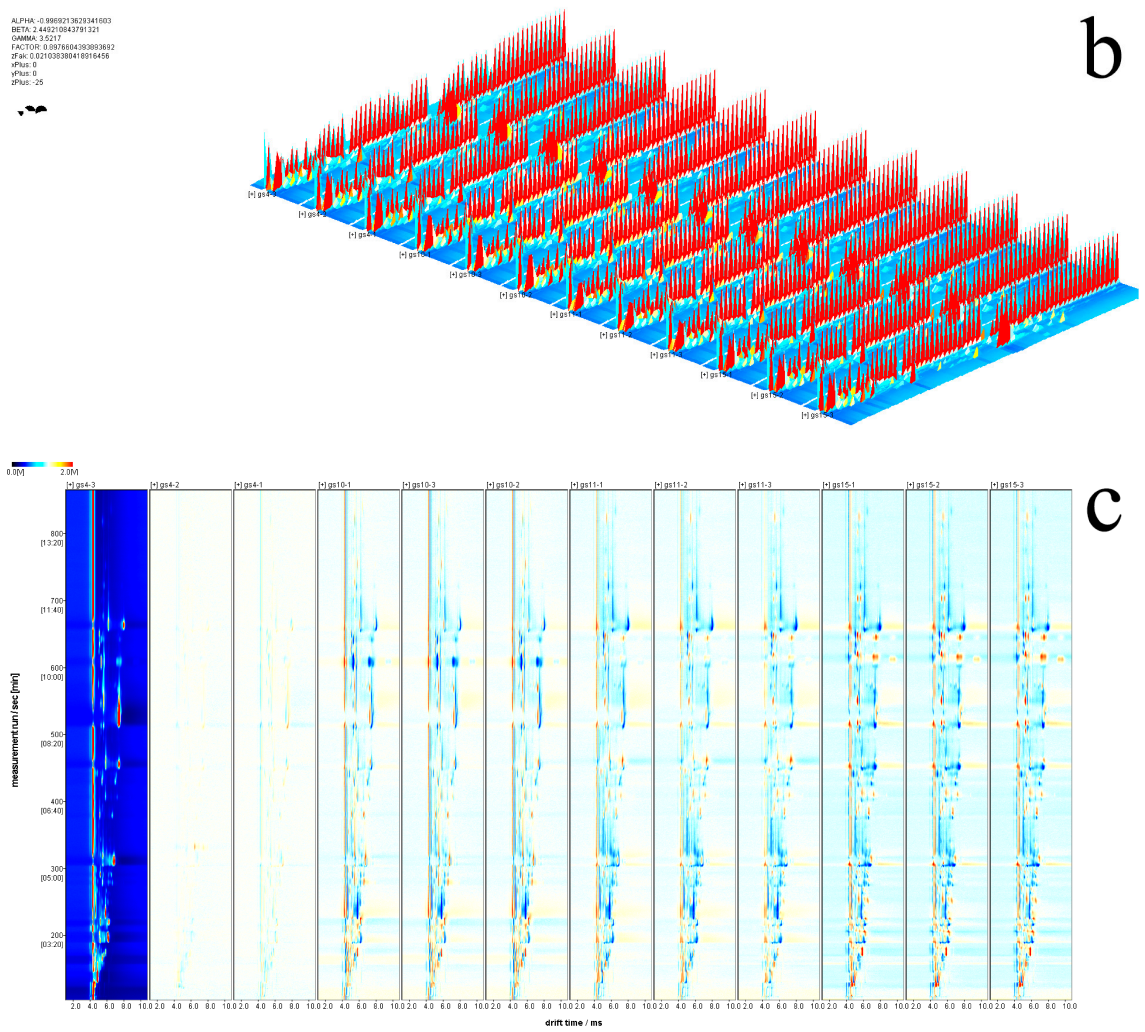
Two-dimensional (**a**), three-dimensional (**b**), and comparative fingerprint map (**c**) of flavor compounds in ginseng powder from different years. Note: gs4-1, gs4-2, gs4-3 are 10-year sample groups; gs10-1, gs10-2, gs10-3 are 15-year sample groups; gs11-1, gs11-2, gs11-3 are 20-year sample groups; gs15-1, gs15-2, gs15-3 are 25-year sample groups.

**Figure 2:**
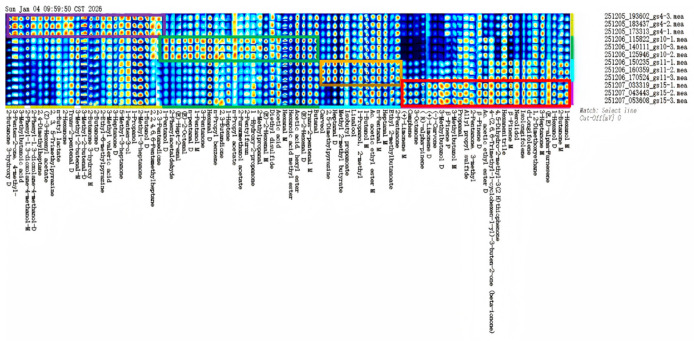
Fingerprint profiles of WG powder from different growth years. Note: The colored boxes are used to mark the differential regions of characteristic volatile compounds in samples.

A total of 68 VOCs were tentatively identified in the samples. According to the classification systems described in food flavor chemistry, these compounds were categorized into 7 major classes based on their functional group structures and odor characteristics (Table 1). Specifically, the tentatively identified compounds comprised seven alcohols, twelve aldehydes, thirteen ketones, ten esters, twelve terpenes, three pyrazines, and eleven other compounds. The three-dimensional, two-dimensional and differential GC-IMS spectra (Figure 1) visually demonstrated enhanced signal intensities with increasing growth years. Notably, the characteristic peaks of the 10-year sample group appeared lighter in color, whereas those of the 25-year sample group were the darkest, indicating a cumulative increase in the content of flavor compounds. Fingerprint maps (Figure 2) demonstrated good reproducibility within the same age group, while clear differences were observed among samples from different age groups.

**Figure 3:**
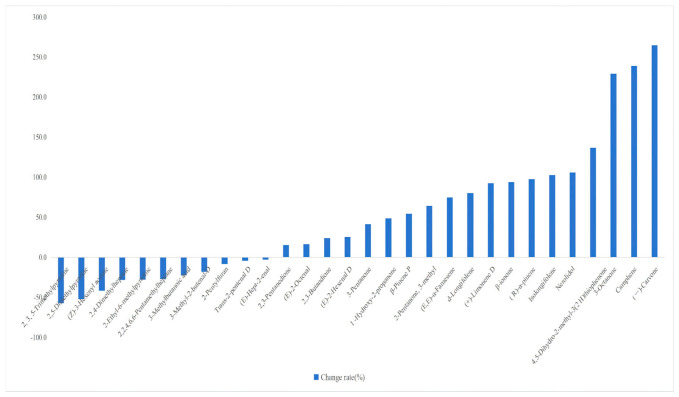
Changes in material composition of WG across different growth years.

Among the 68 compounds, terpenes (12 compounds, accounting for 17.6% of total identifications) and pyrazines (3 compounds, 4.4%) constituted relatively minor fractions in terms of compound numbers (Figure 3). However, these two classes exhibited the most pronounced and contrasting temporal trends across growth years, making them sensitive chemical indicators of WG aging. The remaining 53 compounds (77.9%) displayed more complex, often non-monotonic variation patterns, reflecting the multifaceted regulation of secondary metabolism during WG growth.

### 2.2. Dynamic Changes in Flavor Compounds with Growth Years

**Figure 4:**
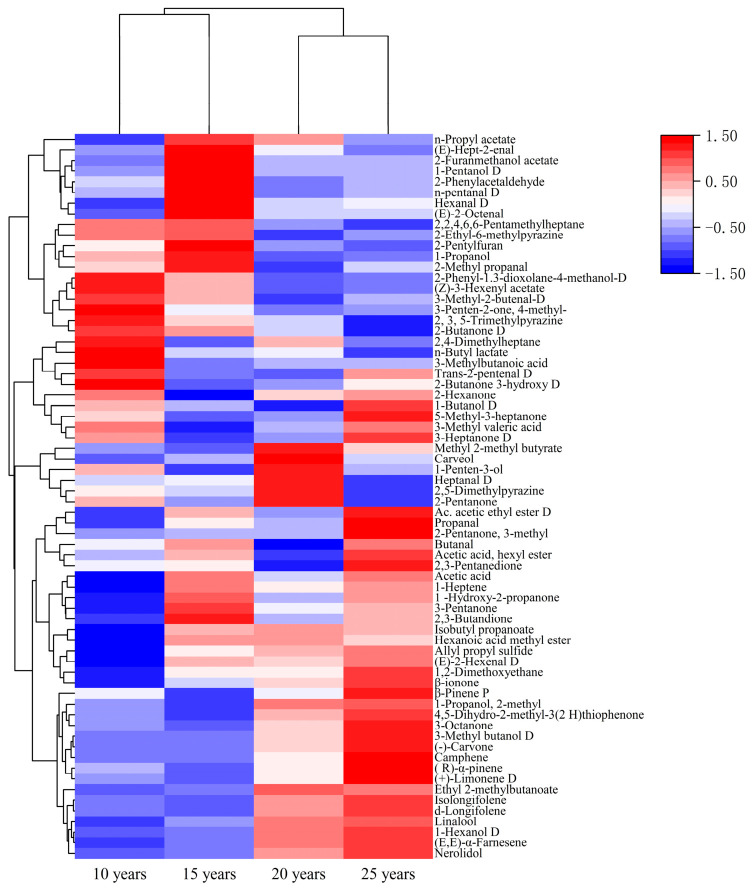
Heatmap of volatile flavor compounds in WG across different growth years.

Heatmap visualization (Figure 4) combined with quantitative data (Table 2) demonstrated the differences in content and the temporal evolution patterns of the 68 key VOCs among the 10-, 15-, 20-, and 25-year groups. The heatmap employed color gradients to clearly distinguish variations in compound abundance. Horizontal clustering indicated a high similarity in VOCs composition among samples within the same growth year, reflecting good intra-group repeatability. Vertical clustering categorized flavor compounds according to their change trends across growth years, thereby revealing the dynamic regulation of secondary metabolism in WG throughout the growth cycle. By integrating the color variations observed in the heatmap with the quantitative statistical data, four distinct evolution patterns were identified. Among these, two contrasting directional trends—the monotonic accumulation of terpenes and the overall decline of pyrazines—emerged as the most prominent chemical signatures of WG aging, while compounds exhibiting increase-then-decrease or fluctuating patterns reflected the complexity and environmental sensitivity of secondary metabolism. Together, these patterns constitute a divergent evolutionary trajectory of WG’s flavor quality.

#### 2.2.1. Terpene Accumulation as a Hallmark of Aging

In the heatmap, compounds exhibiting this pattern displayed progressively darker colors with increasing growth years, and their contents showed a continuous and stable upward trend from 10 to 25 years. These compounds are the primary contributors to the characteristic flavor profile of older WG. Among them, terpenes and their derivatives demonstrated the most pronounced increasing trends, emerging as the dominant drivers of flavor compounds evolution. The sesquiterpenes (E,E)-α-Farnesene and Nerolidol increased steadily from 1.538 mg/kg and 1.551 mg/kg in the 10-year group to 2.686 mg/kg and 3.196 mg/kg in the 25-year group, corresponding to growth rates of 106.1% and 74.6%, respectively. The monoterpene d-Longifolene exhibited a gradual increase from 2.923 mg/kg to 5.27 mg/kg, representing an 80.3% increase, whereas Camphene showed a more pronounced increase, rising from 0.610 mg/kg to 2.068 mg/kg, corresponding to a 239.0% increase. In addition, β-ionone (1.508→2.923 mg/kg) and (−)-Carvone (0.699→2.548 mg/kg) followed the same monotonic increasing pattern, with increases of 93.8% and 264.5%, respectively. Terpene alcohols, including Linalool and 1-Hexanol, also exhibited increases of approximately 50%. This monotonic accumulation pattern directly indicates sustained activity of the mevalonic acid (MVA) and methylerythritol phosphate (MEP) pathways throughout the WG growth cycle, particularly during the later stages, resulting in continuous biosynthesis and accumulation of terpenoid skeleton compounds.

In addition, several aldehydes exhibited progressively darker color intensities in the heatmap. For example, (E)-2-hexenal increased from 1.113 to 1.393 mg/kg, while propanal rose from 1.726 to 3.87 mg/kg, both displaying clear upward trends with increasing growth years. These aldehydes contribute prominently to the fruity and green aroma characteristics of WG.

#### 2.2.2. Divergent Pyrazine Trajectories with a Common Endpoint Decline

In striking contrast to the monotonic terpene accumulation described above, the three pyrazines identified in this study exhibited markedly different temporal patterns yet shared a common feature: their concentrations at 25 years were all substantially lower than at 10 years.

The three compounds followed three distinct trajectories:

2,3,5-Trimethylpyrazine showed a strict monotonic decline, decreasing progressively from 0.735 mg/kg (10 y) to 0.577 mg/kg (15 y), 0.467 mg/kg (20 y), and 0.314 mg/kg (25 y), representing a cumulative reduction of 57.3%.

2-Ethyl-6-methylpyrazine remained relatively stable from 10 to 15 years (0.408 and 0.419 mg/kg, respectively; not significantly different), then declined to 0.263 mg/kg at 20 years, before rising slightly to 0.295 mg/kg at 25 years. Its overall change from 10 to 25 years was a decrease of 27.7%.

2,5-Dimethylpyrazine followed the most complex pattern: it first decreased from 0.449 mg/kg (10 y) to 0.372 mg/kg (15 y), then rose sharply to a maximum of 0.648 mg/kg at 20 years, before declining precipitously to 0.214 mg/kg at 25 years—the lowest concentration recorded for any pyrazine at any stage. Its overall decrease from 10 to 25 years was 52.3%.

Thus, although none of the three pyrazines followed an identical trajectory, all three reached their lowest or near-lowest concentrations at 25 years. The transient elevation of 2,5-dimethylpyrazine at 20 years deviates notably from a simple progressive decline and may reflect a stage-specific metabolic event—possibly involving a temporary shift in amino acid precursor pools or Maillard-type reactions—before the broader attenuation of nitrogen metabolism in the oldest roots. These compounds are commonly associated with roasted and nutty aromas; their overall decline from 10 to 25 years suggests that pathways related to pyrazine formation are ultimately downregulated during WG aging, albeit with compound-specific temporal dynamics. In addition to pyrazines, several ketones and organic acids also exhibited overall decreases, including 2-butanone (decreased after 15 years, reached 1.697 mg/kg at 25 years), 4-methyl-3-penten-2-one (1.04→0.71 mg/kg, remained stable from 20 to 25 years), and 3-methylbutanoic acid (0.17→0.13 mg/kg, stabilized after 15 years), further supporting the broader attenuation of certain nitrogen- and carbonyl-related metabolic pathways in aging WG.

#### 2.2.3. Stage-Specific Metabolic Peaks

Compounds following this pattern exhibited peak contents at 15–20 years, likely corresponding to a period of maximal metabolic activity. This was followed by progressively lighter colors in the heatmap and a decline in content with further aging, reflecting the phase-dependent activation of specific metabolic pathways. For example, the saturated aldehyde Hexanal and the alcohol Carveol reached their highest levels in the 20-year group (5.473 mg/kg and 0.466 mg/kg, respectively). The corresponding regions in the heatmap were darkest at 20 years and gradually lightened with increasing age. The esters Hexanoic acid methyl ester, Ethyl 2-methylbutanoate, and n-Propyl acetate also displayed metabolic peaks during the 15–20-year period. These compounds are likely metabolic intermediates or characteristic products of specific growth stages. Their transient accumulation is indicative of a phase of vigorous physiological metabolism, followed by a reduction in content due to metabolic pathway modulation and the reallocation of metabolic resources. This transient metabolic surge adds a temporal dimension to the divergent evolution model, indicating that certain flavor characteristics are stage-specific rather than linearly age-dependent.

#### 2.2.4. Environmentally Driven Fluctuations

Compounds following this pattern exhibited no clear or consistent color gradient in the heatmap, with their contents fluctuating irregularly across different growth years. Among the 68 tentatively identified volatile components, approximately 66% showed such non-monotonic variation patterns, spanning multiple chemical classes, including alcohols, aldehydes, ketones, esters, etc. The observed fluctuations are likely influenced by annual climatic variability, micro-environmental conditions, or individual genetic differences, rendering these compounds less reliable as independent markers of growth age. The results indicate that the flavor evolution of WG is driven by the integrated effects of multiple compounds rather than by the linear accumulation of all constituents. It reflects the dynamic interplay of distinct metabolic pathways that undergo coordinated activation and attenuation throughout the growth cycle, ultimately shaping the structural evolution of the volatile chemical fingerprint. The predominance of fluctuating changes among the 68 compounds underscores that the divergent evolution of WG flavor is not a simple binary process but a complex, multi-layered phenomenon shaped by both intrinsic developmental programs and extrinsic environmental inputs.

### 2.3. PLS-DA Analysis and Key Biomarker Screening

**Figure 5:**
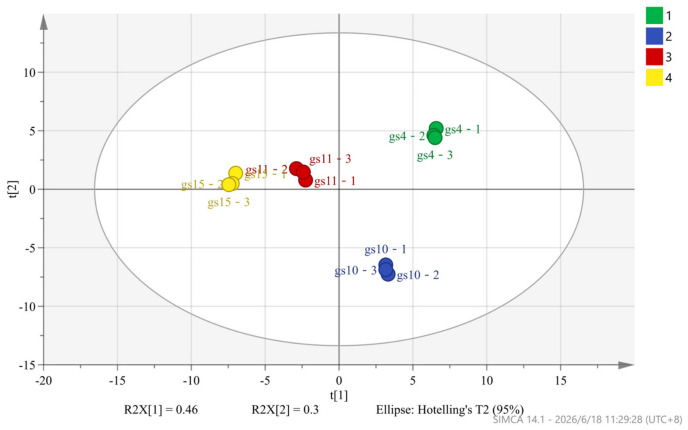
PLS−DA analysis of flavor compounds in WG across different growth years. Note: Green: 10-year group; blue: 15-year group; red: 20-year group; yellow: 25-year group.

**Figure 6:**
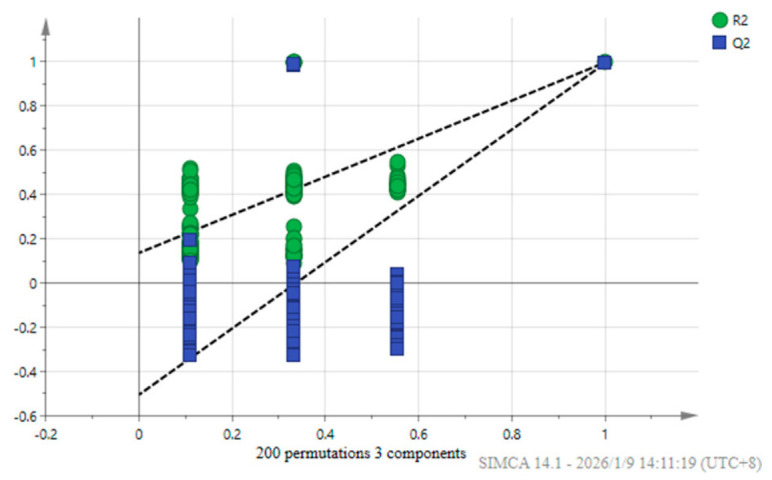
Permutation test results of flavor compounds in WG across different growth years.

The PCA-X preprocessing results showed that R^2^X [1] = 0.467 and R^2^X [2] = 0.298. The first two principal components together explained 76.5% of the total variance in the original data, indicating a satisfactory representation of the dataset. The PLS-DA model (Figure 5) was able to clearly discriminate samples from different growth years, with distinct separation among groups, suggesting pronounced differences in flavor profiles across growth stages. A 200-times permutation test demonstrated the robustness and reliability of the model (Figure 6, R^2^ = 0.136, Q^2^ = −0.505).

**Figure 7:**
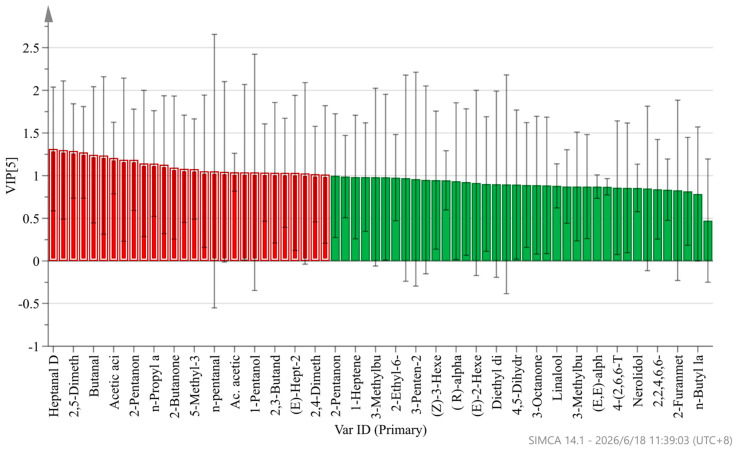
VIP values of the characteristic variables. Note: Red bars denote variables with VIP values > 1.0, while green bars represent those with VIP values < 1.0.

VIP analysis identified 29 differential compounds with VIP > 1.0 (Figure 7), spanning multiple chemical classes including terpenes, aldehydes, ketones, alcohols, esters, pyrazines, and organic acids. The compounds with the highest VIP scores included Heptanal (1.32), 1-butanol (1.32), 2,5-dimethylpyrazine (1.29), 2,3-Pentanedione (1.28), Carveol (1.24), and Butanal (1.24). The complete list of 29 VIP-ranked compounds is provided in Table S1. These compounds can be considered potential chemical biomarkers for discriminating WG samples of different growth years.

## 3. Discussion

A central finding of this study is that the volatile flavor evolution of WG does not follow a uniform trajectory. Instead, it exhibits a divergent pattern: terpenes progressively accumulate while pyrazines progressively decline, with other compounds showing transient peaks or irregular fluctuations. This chemical divergence reflects the differential regulation of metabolic pathways during WG growth, as discussed below.

As a traditional Chinese medicine herb, WG exhibits significantly higher medicinal value than cultivated ginseng [7], and its market value is closely correlated with growth years [11]. However, traditional identification methods based on external morphological features, particularly the “five shapes” criteria [12], although intuitive and practical, fail to objectively reflect the intrinsic quality. At the same time, it constrains the standardized development of deep-processed products. In this study, GC-IMS combined with chemometric methods was employed to systematically elucidate the internal standard method, using 2-methyl-3-heptanone as the reference compound to minimize instrumental variation. The variation patterns of flavor compounds in WG across different growth years at the level of secondary metabolites thereby provide a scientific basis for establishing an objective quality evaluation system for this valuable resource.

### 3.1. Biological Mechanisms Underlying Flavor Compound Evolution in WG

WG grows over long periods in coniferous and broad-leaved mixed forests at altitudes of 400–1000 m, continuously exposed to environmental stresses such as cold, shade, and nutrient competition. The accumulation of secondary metabolites is co-regulated by growth stage and environmental conditions [6,13,14]. The four change patterns observed among the 68 flavor compounds in this study provide evidence for the metabolic response of WG to long-term adaptation to adverse environments. Future work should include GC-MS confirmation or authentic standard verification for key marker compounds.

As defensive metabolites, terpene contents increased continuously with age, which is closely associated with stress-induced activation of the mevalonic acid (MVA) and methylerythritol phosphate (MEP) pathways [15,16]. This finding is consistent with transcriptomic evidence from wild-simulated ginseng, where key genes involved in terpene backbone biosynthesis were progressively upregulated with age [8]. These compounds are not only the primary contributors to the characteristic medicinal aroma of WG but are also consistent with the enhancement of its medicinal activities, such as antioxidant and anti-inflammatory effects. For example, the growth rates of (E,E)-α-farnesene and nerolidol from 10 to 25 years reached 106.5% and 74.6%, respectively, paralleling the reported increases in ginsenoside content in high-age WG [4,17,18]. This provides chemical evidence supporting the traditional experience that “the longer the age, the better the quality.” Compared with other aged plant-based food systems, the monotonic terpene increase in WG shows partial similarity to Pu-er tea during aging, where certain terpenes (e.g., β-caryophyllene, α-copaene) progressively accumulate and contribute to the characteristic “aged aroma” [19]. However, unlike WG, Pu-er tea does not exhibit a concurrent monotonic decline in pyrazines, reflecting differences in microbial involvement and nitrogen metabolism between the two systems. This chemically verifies the traditional experience that “the longer the age, the better the quality.”

In contrast to the monotonic terpene accumulation, the three pyrazines identified in this study followed distinct temporal trajectories, yet all exhibited an overall decrease from 10 to 25 years. 2,3,5-Trimethylpyrazine declined monotonically (57.3% reduction), consistent with a progressive downregulation of its biosynthetic pathway. 2-Ethyl-6-methylpyrazine showed a more moderate decline (27.7%) after an initial plateau. Notably, 2,5-dimethylpyrazine displayed a pronounced transient peak at 20 years before declining sharply to its lowest level at 25 years. This divergence in trajectories, despite a shared endpoint decline, suggests that pyrazine metabolism in aging WG is governed by both a general attenuation of nitrogen metabolism—as resources are increasingly allocated to terpenoid and ginsenoside biosynthesis—and compound-specific regulatory events. The 20-year surge of 2,5-dimethylpyrazine may reflect a temporary shift in amino acid precursor availability or a localized Maillard-type reaction associated with a particular physiological transition, before the broader nitrogen reallocation dominates in the oldest roots. Similar competitive regulation between nitrogen metabolism and phenylpropanoid/terpenoid pathways has been documented in perennial plants [17]. Meanwhile, the “increase-then-decrease” pattern observed for certain aldehydes and esters (e.g., hexanal and hexyl acetate) marks a period of vigorous metabolism in 15–25-year-olds WG. During this stage, physiological activity is at its peak, and the synthesis and transformation rates of secondary metabolites reach their highest levels. Studies on Citri Reticulatae Pericarpium (CRP) have demonstrated that volatile organic compounds evolve from lemon, sweet, and musk aromas to apple, pineapple, and coffee odors during aging, indicating active transformation of flavor-related metabolites throughout the storage process [20]. These parallels suggest that the “increase-then-decrease” pattern may represent a general metabolic feature in aging plant tissues, corresponding to a phase of maximal metabolic activity followed by resource reallocation.

Notably, 66.18% of the flavor compounds exhibited fluctuating changes, closely related to the complexity of the WG growth environment. Non-periodic factors, including annual climate fluctuations, soil microbial community structure, harvest time and precipitation distribution, influence the activity of metabolic pathways involving esters and certain aldehydes and ketones, preventing the formation of stable, time-dependent accumulation patterns [21,22,23]. This finding suggests that a single compound cannot serve as a reliable indicator for age discrimination and underscores the necessity of constructing a multi-marker combination model to improve discrimination accuracy.

### 3.2. Innovation and Reliability of the Technical Methodology

The application of GC-IMS technology in this study fully demonstrates its unique advantages for the analysis of volatile components in complex matrices. Compared with traditional GC-MS, GC-IMS requires no complex sample pretreatment, exhibits higher sensitivity for detecting short-chain and low-concentration volatile components, and generates fingerprints with good repeatability. The characteristic peak distributions of samples from the same growth year were highly consistent, providing solid technical support for the establishment of WG chemical fingerprints [22]. The integrated technical framework of “retention index qualification + internal standard semi-quantification + PLS-DA discrimination” adopted in this study further enhanced the reliability of the analytical results. In the PLS-DA model, the first two principal components cumulatively explained 76.5% of the variation. The PLS-DA model exhibited strong explanatory and predictive capacity, with R^2^X = 0.948, R^2^Y = 0.997, and Q^2^ = 0.993. The high Q^2^ value (close to 1) indicates excellent model predictive ability. To rigorously exclude the possibility of overfitting, a 200-times permutation test was conducted. In this test, the Q^2^ intercept was −0.505 (below zero), and the R^2^ intercept was 0.136. These results satisfy the generally accepted criterion that a Q^2^ intercept ≤ 0.05 (or negative) indicates no overfitting, confirming that the model could effectively distinguish samples from different growth years. Furthermore, the 29 differential compounds (VIP > 1) tentatively identified through VIP analysis covered key metabolic pathway products of WG, and their combined discriminative performance was markedly superior to that of any single indicator.

### 3.3. Limitations and Future Directions

Several limitations of this study should be acknowledged. First, although the three sampling sites were matched for altitude, slope aspect, and vegetation type to minimize environmental heterogeneity, soil chemistry, microclimatic variables, and rhizosphere microbial communities were not quantified. Some of the observed VOC variation may therefore be attributable to unmeasured micro-environmental differences. Second, the sample size of three site-level composite replicates per age group, while representing independent spatial replicates, remains limited. Third, the pooling strategy, while intentional to capture population-level VOC characteristics, precludes assessment of individual-level variability. Fourth, as is standard practice in non-targeted HS-GC-IMS profiling, all compound identities in this study are tentative, based on retention index (RI) and drift time (Dt) matching against the instrument’s built-in database, which was originally constructed using authentic standards. While the dual-parameter (RI + Dt) matching approach provides reasonable confidence for exploratory screening, definitive structural confirmation of the proposed markers with authentic standards would further strengthen the discrimination framework. Future studies should therefore incorporate targeted verification of key markers, alongside individual-root analyses with larger sample sizes, multi-region sampling, and soil and microclimate monitoring.

## 4. Materials and Methods

### 4.1. Samples and Sampling Design

**Figure 8:**
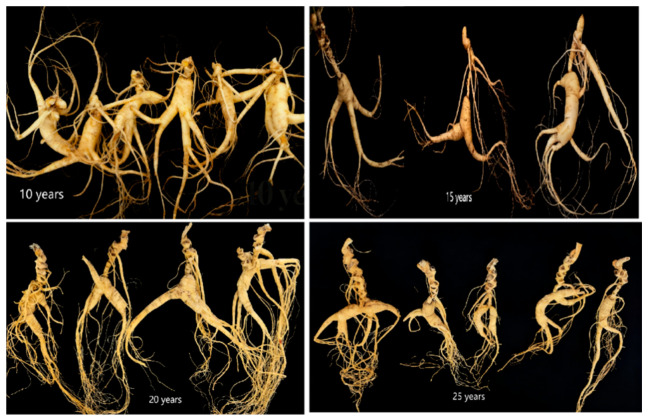
Partial wild ginseng samples collected from Huanren County, Liaoning Province, in the study.

Wild ginseng (WG) samples (Figure 8) were collected from three sites (S1, S2, S3) within Hengdaohezi Village, Huanren County, Liaoning Province, China (Table 3). All samples were independently authenticated by expert appraisers from the Quality Inspection and Testing Center for Special Economic Animal and Plant Products, Ministry of Agriculture and Rural Affairs. Growth year was determined following the national standard GB/T 18765-2015 [24] Identification and Grading Quality of Wild Ginseng. Age identification was based on three complementary criteria: (1) counting of rhizome bud scars, which form annually at the base of the aerial stem and provide the primary chronological record; (2) examination of scar trace clusters on the smooth rhizome portion, particularly for older specimens in which individual bud scars become compressed and indistinct; and (3) comprehensive assessment of periderm color and texture, which coarsens and darkens progressively with increasing age. All age assignments were made prior to and independently of any chemical analysis.

The three sites were selected for similar altitude (443–485 m), slope aspect (northeast), and vegetation type (coniferous and broad-leaved mixed forest) to minimize environmental heterogeneity. At each site, for each of the four growth-year cohorts (10, 15, 20, and 25 years), a minimum of five intact WG roots were collected and pooled to obtain one site-representative composite sample. This yielded three independent site-level composite samples per age group (3 sites × 4 age groups = 12 composite samples), with all cohorts collected on the same date (26 August 2025), constituting a balanced site × age design.

Sample codes were assigned as: gs4 = 10-year, gs10 = 15-year, gs11 = 20-year, gs15 = 25-year; the numerical suffix denotes the site (e.g., gs4-1 = 10-year sample from S1).

The pooling strategy was intentionally employed to minimize inter-individual variability and capture the population-level VOC characteristics of each age cohort, thereby enhancing the robustness and generalizability of the chemometric models.

After collection, all samples were washed, dried at 50 °C, pulverized, sieved through an 80-mesh sieve, homogenized, and stored at −20 °C until analysis.

GC-IMS calibration standards (C4-C9 n-ketone) were purchased from Aladdin Co., Ltd. (Shanghai, China). The internal standard, 2-methyl-3-heptanone (CAS No. 13019-20-0, Lot No. 25070276, purity 99.5%), was obtained from Tan-Mo Technology Co., Ltd. (Changzhou, China). High-purity nitrogen (≥99.999%) was used as the carrier gas, and all other reagents were of analytical grade.

### 4.2. Instruments

FlavourSpec^®^ gas chromatography-ion mobility spectrometer (G.A.S. GmbH, Dortmund, Germany) was used, equipped with a CTC PAL3 automatic headspace sampler (CTC Analytics AG, Zwingen, Switzerland), an MXT-5 capillary column (15 m × 0.53 mm, 1.0 µm, Restek Corporation, Bellefonte, PA, USA), and VOCal data processing software (version 0.4.03, G.A.S. GmbH, Dortmund, Germany).

### 4.3. HS-GC-IMS Analysis

VOCs were analyzed by HS-GC-IMS following the method of Zou et al. (2025) and Xiang et al. (2024) [25,26] with minor modifications. Powdered sample (0.5000 g) was placed in a 20 mL headspace vial with 5 µL of internal standard (2500 µg/mL), then incubated at 70 °C for 20 min under agitation at 500 rpm. Headspace gas (500 µL) was injected using an 85 °C syringe. GC separation employed an MXT-5 column at 60 °C with high-purity N_2_ as carrier gas. The flow program: 2.0 mL/min (2 min)→10.0 mL/min (8 min)→100.0 mL/min (10 min)→150.0 mL/min (10 min), held 30 min; injector, 80 °C. IMS detection used N_2_ drift gas (75 mL/min) through a 53 mm drift tube (45 °C, 500 V/cm), with a ^3^H ionization source in positive ion mode.

### 4.4. Qualitative and Semi-Quantitative Estimates

Compounds were tentatively identified by matching retention index (RI) calculated from n-ketone standards and drift time with databases the NIST 2020 library and the IMS databases [24]. Semi-quantification was carried out using an internal standard method, with 2-methyl-3-heptanone as the internal standard. The content of each target compound (mg/kg) was calculated according to the following formula:

$$W_{x}=\frac{C_{\mathrm{is}}\times V_{\mathrm{is}}}{1000\times m}\times \frac{A_{x}}{A_{\mathrm{is}}}$$

where W_x_ represents the content of the target compound, C_is_ is the concentration of the internal standard (µg/mL), V_is_ is the volume of internal standard added (µL), m is the sample weight (g), and A_x_ and A_is_ are the peak areas of the target compound and the internal standard, respectively.

### 4.5. Data Processing and Statistical Analysis

GC-IMS data were processed using the Reporter and Gallery Plot plugins in VOCal software (version 0.4.03). Prior to analysis of variance (ANOVA), data normality was assessed using the Shapiro–Wilk test, and homogeneity of variances was verified by Levene’s test. One-way ANOVA was performed to evaluate differences among the four growth-year groups for each compound. Statistical analysis was performed using one-way analysis of variance (ANOVA) with SPSS software (version 26.0). When significant differences were detected (*p* < 0.05), post hoc multiple comparisons were conducted using Tukey’s HSD test; results are expressed as mean ± standard error (SE). PLS-DA and VIP analysis were conducted using SIMCA software (version 14.1). PLS-DA model validation was performed using a 200-times permutation test. Heatmaps were generated using Origin software (version 2025b).

## 5. Conclusions

This study reveals that the VOC evolution of WG across 10–25 growth years follows a divergent trajectory: terpenes progressively accumulate, while pyrazines exhibit an overall decline from 10 to 25 years through distinct compound-specific trajectories—2,3,5-trimethylpyrazine declines monotonically (57.3% reduction), 2-ethyl-6-methylpyrazine shows a moderate overall decrease (27.7%) after an initial plateau, and 2,5-dimethylpyrazine undergoes a pronounced transient peak at 20 years before a sharp terminal decline (52.3% overall reduction). These divergent pyrazine dynamics indicate that nitrogen metabolism in aging WG is governed by both a general attenuation trend and compound-specific regulatory events. The majority of remaining VOCs (66%) displayed non-monotonic patterns, including stage-specific metabolic peaks and environmentally driven fluctuations, underscoring the multifaceted nature of VOC remodeling during WG growth.

HS-GC-IMS combined with PLS-DA enabled the tentative identification of 68 VOCs and 29 differential markers (VIP > 1), with the model effectively discriminating samples across growth years. Preliminary reference thresholds derived from 95% confidence interval limits were proposed for three growth phases (10–15, 15–20, and 20–25 years), providing a chemical framework for flavor-based growth-year assessment that complements traditional morphology-based identification.

It should be noted that the proposed marker combinations and thresholds, derived from tentatively identified compounds in pooled site-representative samples, represent a testable framework rather than a validated quality control tool. The sampling design—pooling multiple roots per site to capture population-level VOC characteristics—enhances the generalizability of the chemometric models for batch-level assessment, though individual-root validation remains necessary. All compound identities are tentative, based on dual-parameter (RI + Dt) database matching, and require future confirmation with authentic standards.

This study provides a chemical basis for the objective quality evaluation of WG powder, with particular relevance to “high-quality but poor-shape” specimens that are systematically undervalued by conventional morphology-based grading. Future studies should: (1) validate the proposed markers using individual-root samples with sufficient biological replicates and optimize cutoff values through ROC analysis; (2) incorporate multi-region sampling across broader geographic and environmental gradients; (3) confirm the identities of key marker compounds with authentic standards; and (4) integrate GC-O and sensory evaluation to bridge the gap between chemical fingerprints and human aroma perception.

## Figures and Tables

**Table 1:** Results of the qualitative analysis of different growth years of wild ginseng.

Category	Compound	CAS#	Formula	MW	RI	Rt/s	Dt/ms	Odor Description
Terpenes	Nerolidol	C7212444	C_15_H_26_O	222.4	1541.1	2337.605	1.49178	flower, green, waxy, citrus aroma, woody flavor
	(E, E)-α-Farnesene	C502614	C_15_H_24_	204.4	1518.8	2217.096	1.43251	citrus herbal lavender bergamot myrrh neroli green
	d-Longifolene	C475207	C_15_H_24_	204.4	1407.4	1702.666	1.44243	sweet woody rose medical fir needle
	Iso-Longifolene	C1135666	C_15_H_24_	204.4	1371.9	1565.467	1.44243	woody
	(+)-Limonene M	C138863	C_10_H_16_	136.2	1033.9	702.724	1.21679	lemon, sweet, orange, pine oil
	(+)-Limonene D	C138863	C_10_H_16_	136.2	1034.1	703.113	1.29548	lemon, sweet, orange, pine oil
	Camphene	C79925	C_10_H_16_	136.2	954.5	550.744	1.209	woody, camphor
	(R)-α-pinene	C7785708	C_10_H_16_	136.2	937.1	516.454	1.21259	Terpenic, Mint, Pine
	β-Pinene M	C127913	C_10_H_16_	136.2	983.7	613.341	1.21737	resin, green
	β-Pinene D	C127913	C_10_H_16_	136.2	984.5	615.313	1.29282	resin, green
	β-Pinene P	C127913	C_10_H_16_	136.2	983.8	613.67	1.72327	resin, green
	β-ionone	C14901076	C_13_H_20_O	192.3	1499	2115.677	1.47531	rose, floral, iris, fruity, woody
	(−)-Carvone	C99490	C_10_H_14_O	150.2	1246.6	1163.382	1.31536	spearmint
	Carveol	C99489	C_10_H_16_O	152.2	1217.7	1086.292	1.29358	fresh, spearmint, caraway
	Linalool	C78706	C_10_H_18_O	154.3	1100.2	822.177	1.25066	citrus, rose, woody, blueberry
Ketones	3-Octanone	C106683	C_8_H_16_O	128.2	998.1	645.59	1.70914	moldy, ketone, green, waxy, vegetable, mushroom, fruity
	3-Heptanone M	C106354	C_7_H_14_O	114.2	890.2	434.486	1.23525	Fruity, Grass, Oil
	3-Heptanone D	C106354	C_7_H_14_O	114.2	889	432.685	1.58984	Fruity, Grass, Oil
	2-Hexanone	C591786	C_6_H_12_O	100.2	804.6	323.427	1.19587	fruity, fungal, meaty, buttery
	2-butanone 3-hydroxy M	C513860	C_4_H_8_O_2_	88.1	727.6	246.218	1.06235	butter, cream
	2-butanone 3-hydroxy D	C513860	C_4_H_8_O_2_	88.1	719.2	238.956	1.33411	butter, cream
	2-Pentanone	C107879	C_5_H_10_O	86.1	689.2	214.626	1.11425	acetone, fresh, sweet fruity, wine
	2,3-Pentanedione	C600146	C_5_H_8_O_2_	100.1	714.5	235.015	1.22865	sweet, cream, caramel, nuts, cheese
	2,3-butanedione	C431038	C_4_H_6_O_2_	86.1	589.3	166.606	1.17706	butter, popcorn, sweet taste, sour rice
	2-Butanone D	C78933	C_4_H_8_O	72.1	596.9	169.852	1.24222	fruity, camphor
	2-Butanone M	C78933	C_4_H_8_O	72.1	596.9	169.852	1.05683	fruity, camphor
	3-Pentanone	C96220	C_5_H_10_O	86.1	694.2	218.547	1.35976	ethereal
	2-pentanone, 3-methyl	C565617	C_6_H_12_O	100.2	747.8	264.748	1.46842	mint, honey
	4-methyl-3-Penten-2-one	C141797	C_6_H_10_O	98.1	797.3	315.419	1.43959	spice, earth, green
	1-Hydroxy-2-propanone	C116096	C_3_H_6_O_2_	74.1	685.5	212.523	1.04191	pungent, caramel, fresh
Aldehydes	(E)-2-Octenal	C2548870	C_8_H_14_O	126.2	1064.1	754.849	1.33015	fresh cucumber, fatty, green herbal, banana, green leaf
	(E)-Hept-2-enal	C18829555	C_7_H_12_O	112.2	964.5	571.404	1.65754	spicy, green vegetables, fresh, fatty
	(E)-2-Hexenal M	C6728263	C_6_H_10_O	98.1	854.2	383.795	1.17996	green, banana, fat
	(E)-2-Hexenal D	C6728263	C_6_H_10_O	98.1	852.8	381.993	1.51532	green, banana, fat
	3-Methyl-2-butenal D	C107868	C_5_H_8_O	84.1	784.8	302.125	1.35719	fruity
	3-Methyl-2-butenal M	C107868	C_5_H_8_O	84.1	783.9	301.197	1.09391	fruity
	Trans-2-pentenal M	C1576870	C_5_H_8_O	84.1	754.6	271.19	1.1033	potato, peas
	Trans-2-pentenal D	C1576870	C_5_H_8_O	84.1	753	269.656	1.36007	potato, peas
	Heptanal M	C111717	C_7_H_14_O	114.2	906.1	460.475	1.33381	fresh, aldehyde, fatty, green herbs, wine, fruity
	Heptanal D	C111717	C_7_H_14_O	114.2	905	458.674	1.68961	fresh, aldehyde, fatty, green herbs, wine, fruity
	Hexanal M	C66251	C_6_H_12_O	100.2	802.9	321.554	1.26234	fresh, green, fat, fruity
	Hexanal D	C66251	C_6_H_12_O	100.2	797.3	315.419	1.56223	fresh, green, fat, fruity
	Butanal	C123728	C_4_H_8_O	72.1	606.5	174.038	1.28922	pungent, fruity, green leaf
	Propanal	C123386	C_3_H_6_O	58.1	493.6	130.788	1.05642	pungent, green grassy
	n-Pentanal M	C110623	C_5_H_10_O	86.1	699.2	222.482	1.19633	green grassy, faint banana, pungent
	n-Pentanal D	C110623	C_5_H_10_O	86.1	698.9	222.224	1.4229	green grassy, faint banana, pungent
	2-Methyl propanal	C78842	C_4_H_8_O	72.1	571.4	159.218	1.28734	banana, melon, slightly nutty
	2-Phenylacetaldehyde	C122781	C_8_H_8_O	120.2	1048.5	727.397	1.25277	hyacinth, sweet fruity, almond, cherry, clover honey, cocoa
Alcohols	1-Hexanol M	C111273	C_6_H_14_O	102.2	874.3	411.328	1.3242	fresh, fruity, wine, sweet, green
	1-Hexanol D	C111273	C_6_H_14_O	102.2	873.4	410.041	1.64033	fresh, fruity, wine, sweet, green
	1-Pentanol M	C71410	C_5_H_12_O	88.1	765.7	282.183	1.25372	balsamic
	1-Pentanol D	C71410	C_5_H_12_O	88.1	766.2	282.695	1.51337	balsamic
	1-Butanol M	C71363	C_4_H_10_O	74.1	664.8	201.668	1.18155	wine
	1-Butanol D	C71363	C_4_H_10_O	74.1	661.8	200.144	1.37446	wine
	1-Propanol	C71238	C_3_H_8_O	60.1	533.1	144.55	1.11957	alcohol, pungent
	3-Methyl butanol M	C123513	C_5_H_12_O	88.1	734.9	252.73	1.23831	whiskey, banana, fruity
	3-Methyl butanol D	C123513	C_5_H_12_O	88.1	735.1	252.98	1.49052	whiskey, banana, fruity
	1-Propanol, 2-methyl	C78831	C_4_H_10_O	74.1	630.3	184.825	1.17479	fresh, alcoholic, leather
	1-Penten-3-ol	C616251	C_5_H_10_O	86.1	686.7	213.184	0.93418	ethereal, green, tropical fruity
Esters	Acetic acid, hexyl ester	C142927	C_8_H_16_O_2_	144.2	1012.4	667.852	1.40698	fruity, green, apple, banana, sweet
Esters	Hexanoic acid methyl ester	C106707	C_7_H_14_O_2_	130.2	937.2	516.526	1.27887	pineapple, apricot, fruity
	Isobutyl propanoate	C540421	C_7_H_14_O_2_	130.2	863.6	396.403	1.2641	rum, pineapple
	Ethyl 2-methyl butanoate	C7452791	C_7_H_14_O_2_	130.2	857.5	388.169	1.23645	apple
	Methyl 2-methyl butyrate	C868575	C_6_H_12_O_2_	116.2	777.6	294.455	1.18953	apple
	Ac. acetic ethyl ester M	C141786	C_4_H_8_O_2_	88.1	619.7	179.945	1.09967	fresh, fruity, sweet, grassy
	Ac. acetic ethyl ester D	C141786	C_4_H_8_O_2_	88.1	618.5	179.373	1.33521	fresh, fruity, sweet, grassy
	n-Propyl acetate	C109604	C_5_H_10_O_2_	102.1	713.7	234.268	1.16076	fruity, pear
	(Z)-3-Hexenyl acetate	C3681718	C_8_H_14_O_2_	142.2	1012	667.231	1.82102	fresh green grassy, sweet, fruity, banana
	n-Butyl lactate	C34451199	C_7_H_14_O_3_	146.2	1019.8	679.633	1.26842	sweet, fruity
	2-Furanmethanol acetate	C623176	C_7_H_8_O_3_	140.1	992.9	634.545	1.42368	sweet, banana
Pyrazines	2,3,5-Trimethylpyrazine	C14667551	C_7_H_10_N_2_	122.2	1003.7	654.171	1.16189	roasted potato, peanut, cocoa, chocolate
	2-Ethyl-6-methylpyrazine	C13925036	C_7_H_10_N_2_	122.2	994.8	639.097	1.17084	nutty, roast, sweet
	2,5-Dimethylpyrazine	C123320	C_6_H_8_N_2_	108.1	896.5	444.522	1.10784	nutty, peanut, moldy, earthy, potato, fatty, cocoa powder
Others	1,2-Dimethoxyethane	C110714	C_4_H_10_O_2_	90.1	655.2	196.816	1.30428	ether
	Allyl propyl sulfide	C27817670	C_6_H_12_S	116.2	873.8	410.567	1.39721	garlic, onion
	2-Pentylfuran	C3777693	C_9_H_14_O	138.2	999.1	647.111	1.25551	bean, fruity, earthy, green, vegetable
	4,5-Dihydro-2-methyl-3(2 H)thiophenone	C13679851	C_5_H_8_OS	116.2	998.4	646.054	1.21561	cabbage, onion, must
	2-Phenyl-1,3-dioxolane-4-methanol M	C1708390	C_10_H_12_O_3_	180.2	967.8	578.487	1.14923	sweet berries, bitter almonds
	2-Phenyl-1.3-dioxolane-4-methanol D	C1708390	C_10_H_12_O_3_	180.2	967.3	577.271	1.46389	sweet berries, bitter almonds
	2,2,4,6,6-Pentamethylheptane	C13475826	C_12_H_26_	170.3	990.3	628.653	1.15566	tasteless
	2,4-Dimethylheptane	C2213232	C_9_H_20_	128.3	824.5	346.427	1.20149	gasoline
	1-Heptene	C592767	C_7_H_14_	98.2	687.3	213.482	1.07836	gasoline
	3-Methyl valeric acid	C105431	C_6_H_12_O_2_	116.2	945.5	532.616	1.59647	sour, herbal, slight green
	Acetic acid	C64197	C_2_H_4_O_2_	60.1	633.7	186.424	1.04583	spicy
	3-Methylbutanoic acid	C503742	C_5_H_10_O_2_	102.1	875.2	412.65	1.48565	sour, foot sweat, cheese

Note: The suffixes M, D, or P denote the monomer, dimer, and polymer forms of the same compound. Odor descriptions were primarily obtained from the database of https://www.flavornet.org (accessed on 15 December 2025).

**Table 2:** Semi-quantitative concentrations of flavor compounds in WG from different growth years.

Compounds	Semi-Quantitative Concentrations (Mean ± Standard Error [SE], mg/kg)			
	10-Year Group	15-Year Group	20-Year Group	25-Year Group
Nerolidol	1.551 ± 0.016 ^d^	1.763 ± 0.002 ^c^	2.695 ± 0.019 ^b^	3.196 ± 0.017 ^a^
(E,E)-α-Farnesene	1.538 ± 0.011 ^d^	1.735 ± 0.02 ^c^	2.495 ± 0.033 ^b^	2.686 ± 0.019 ^a^
d-Longifolene	2.923 ± 0.024 ^d^	2.806 ± 0.024 ^c^	4.642 ± 0.006 ^b^	5.27 ± 0.013 ^a^
Isolongifolene	1.605 ± 0.019 ^c^	1.566 ± 0.014 ^c^	2.871 ± 0.05 ^b^	3.254 ± 0.037 ^a^
(+)-Limonene D	0.134 ± 0.001 ^d^	0.108 ± 0.002 ^c^	0.172 ± 0.001 ^b^	0.258 ± 0.003 ^a^
Camphene	0.61 ± 0.014 ^c^	0.54 ± 0.002 ^c^	1.214 ± 0.012 ^b^	2.068 ± 0.05 ^a^
(R)-α-pinene	0.841 ± 0.009 ^d^	0.578 ± 0.002 ^c^	1.056 ± 0.028 ^b^	1.66 ± 0.042 ^a^
β-Pinene P	1.027 ± 0.007 ^b^	0.619 ± 0.005 ^c^	1.075 ± 0.032 ^b^	1.586 ± 0.011 ^a^
β-ionone	1.508 ± 0.008 ^d^	2.081 ± 0.021 ^c^	2.416 ± 0.004 ^b^	2.923 ± 0.037 ^a^
(−)-Carvone	0.699 ± 0.01 ^d^	0.767 ± 0.008 ^c^	1.614 ± 0.021 ^b^	2.548 ± 0.006 ^a^
3-Octanone	0.258 ± 0.006 ^d^	0.123 ± 0.004 ^c^	0.547 ± 0.027 ^b^	0.849 ± 0.009 ^a^
3-Heptanone D	0.761 ± 0.021 ^a^	0.647 ± 0.012 ^b^	0.676 ± 0.024 ^b^	0.785 ± 0.008 ^a^
2-Hexanone	0.126 ± 0.002 ^a^	0.06 ± 0.001 ^c^	0.112 ± 0.003 ^b^	0.12 ± 0.003 ^ab^
2-Butanone 3-hydroxy D	5.214 ± 0.063 ^d^	1.721 ± 0.009 ^c^	2.196 ± 0.023 ^b^	3.234 ± 0.033 ^a^
2-Pentanone	0.239 ± 0.002 ^a^	0.224 ± 0.004 ^b^	0.253 ± 0.006 ^a^	0.216 ± 0.002 ^b^
2,3-Pentanedione	0.344 ± 0.003 ^b^	0.353 ± 0.004 ^b^	0.304 ± 0.003 ^c^	0.396 ± 0.002 ^a^
2,3-Butandione	0.424 ± 0.006 ^d^	0.584 ± 0.004 ^a^	0.475 ± 0.005 ^c^	0.525 ± 0.012 ^b^
2-Butanone D	2.25 ± 0.049 ^a^	2.121 ± 0.044 ^a^	1.931 ± 0.032 ^b^	1.697 ± 0.065 ^c^
3-Pentanone	0.082 ± 0.006 ^c^	0.129 ± 0.001 ^a^	0.106 ± 0.002 ^b^	0.116 ± 0.001 ^b^
2-Pentanone, 3-methyl	0.014 ± 0.001 ^b^	0.015 ± 0 ^b^	0.015 ± 0.001 ^b^	0.023 ± 0 ^a^
3-Penten-2-one, 4-methyl	1.042 ± 0.013 ^a^	0.805 ± 0.006 ^b^	0.711 ± 0.011 ^c^	0.731 ± 0.007 ^c^
5-Methyl-3-heptanone	2.846 ± 0.033 ^ab^	2.695 ± 0.023 ^c^	2.74 ± 0.027 ^bc^	2.951 ± 0.027 ^a^
1-Hydroxy-2-propanone	0.405 ± 0.007 ^a^	0.638 ± 0.004 ^c^	0.491 ± 0.006 ^b^	0.602 ± 0.005 ^d^
(E)-2-Octenal	0.197 ± 0.003 ^c^	0.331 ± 0.007 ^a^	0.227 ± 0.004 ^b^	0.229 ± 0.002 ^b^
(E)-Hept-2-enal	0.319 ± 0.008 ^c^	0.462 ± 0.006 ^a^	0.351 ± 0.007 ^b^	0.309 ± 0.004 ^c^
(E)-2-Hexenal D	1.113 ± 0.009 ^c^	1.337 ± 0.013 ^b^	1.321 ± 0.009 ^b^	1.393 ± 0.016 ^a^
3-Methyl-2-butenal D	1.598 ± 0.022 ^a^	1.471 ± 0.007 ^b^	1.191 ± 0.023 ^d^	1.306 ± 0.016 ^c^
Trans-2-pentenal D	0.181 ± 0.003 ^a^	0.157 ± 0.002 ^b^	0.156 ± 0.001 ^b^	0.173 ± 0.003 ^a^
Heptanal D	3.984 ± 0.025 ^b^	4.127 ± 0.138 ^b^	5.473 ± 0.102 ^a^	3.058 ± 0.049 ^c^
Hexanal D	7.341 ± 0.045 ^d^	9.312 ± 0.045 ^b^	7.947 ± 0.025 ^c^	8.099 ± 0.014 ^a^
Butanal	0.859 ± 0.006 ^b^	0.92 ± 0.007 ^ab^	0.735 ± 0.033 ^c^	0.94 ± 0.008 ^a^
Propanal	1.726 ± 0.03 ^d^	2.703 ± 0.035 ^b^	2.307 ± 0.026 ^c^	3.87 ± 0.034 ^a^
n-Pentanal D	1.723 ± 0.01 ^b^	2.557 ± 0.014 ^a^	1.571 ± 0.011 ^c^	1.752 ± 0.01 ^b^
2-Methylpropanal	0.118 ± 0.006 ^ab^	0.129 ± 0.004 ^a^	0.103 ± 0.006 ^b^	0.112 ± 0.006 ^ab^
2-Phenylacetaldehyde	0.128 ± 0.003 ^b^	0.205 ± 0.002 ^a^	0.11 ± 0.003 ^c^	0.125 ± 0.003 ^b^
Carveol	0.228 ± 0.006 ^c^	0.283 ± 0.006 ^b^	0.466 ± 0.01 ^a^	0.296 ± 0.002 ^b^
Linalool	0.373 ± 0.006 ^c^	0.415 ± 0.002 ^b^	0.542 ± 0.01 ^a^	0.561 ± 0.005 ^a^
1-Hexanol D	0.875 ± 0.004 ^d^	0.936 ± 0.007 ^c^	1.235 ± 0.022 ^b^	1.308 ± 0.011 ^a^
1-Pentanol D	0.179 ± 0.003 ^b^	0.346 ± 0.003 ^a^	0.187 ± 0.002 ^b^	0.19 ± 0.004 ^b^
1-Butanol D	5.624 ± 0.06 ^b^	5.025 ± 0.021 ^c^	4.488 ± 0.055 ^d^	6.065 ± 0.02 ^a^
1-Propanol	13.511 ± 0.152 ^b^	14.136 ± 0.104 ^a^	12.621 ± 0.086 ^c^	12.743 ± 0.021 ^c^
3-Methyl butanol D	0.032 ± 0 ^c^	0.031 ± 0.001 ^c^	0.095 ± 0.004 ^b^	0.158 ± 0.003 ^a^
1-Propanol, 2-methyl	0.144 ± 0.002 ^c^	0.129 ± 0.001 ^d^	0.181 ± 0.003 ^b^	0.19 ± 0.002 ^a^
1-Penten-3-ol	0.394 ± 0.003 ^b^	0.358 ± 0.002 ^d^	0.415 ± 0.003 ^a^	0.373 ± 0.001 ^c^
Acetic acid, hexyl ester	2.785 ± 0.033 ^c^	2.905 ± 0.006 ^b^	2.682 ± 0.024 ^d^	3.004 ± 0.019 ^a^
Ac. acetic ethyl ester D	1.121 ± 0.029 ^d^	2.214 ± 0.062 ^b^	1.49 ± 0.068 ^c^	2.872 ± 0.034 ^a^
Hexanoic acid methyl ester	1.067 ± 0.014 ^b^	1.271 ± 0.008 ^a^	1.267 ± 0.01 ^a^	1.231 ± 0.008 ^a^
Isobutyl propanoate	0.114 ± 0.002 ^b^	0.15 ± 0.002 ^a^	0.151 ± 0.002 ^a^	0.15 ± 0.002 ^a^
Ethyl 2-methylbutanoate	0.168 ± 0.002 ^d^	0.188 ± 0.001 ^b^	0.31 ± 0.003 ^a^	0.294 ± 0.005 ^c^
Methyl 2-methyl butyrate	1.078 ± 0.006 ^c^	0.994 ± 0.014 ^d^	1.434 ± 0.005 ^a^	1.214 ± 0.012 ^b^
n-Propyl acetate	0.078 ± 0.005 ^d^	0.172 ± 0.002 ^a^	0.154 ± 0.003 ^b^	0.105 ± 0.004 ^c^
(Z)-3-Hexenyl acetate	3.144 ± 0.039 ^a^	2.543 ± 0.031 ^b^	1.692 ± 0.02 ^d^	1.835 ± 0.038 ^c^
n-Butyl lactate	0.173 ± 0.004 ^a^	0.158 ± 0.004 ^b^	0.16 ± 0.002 ^ab^	0.151 ± 0.004 ^b^
2-Furanmethanol acetate	0.042 ± 0.002 ^a^	0.05 ± 0.002 ^a^	0.043 ± 0.001 ^a^	0.043 ± 0.001 ^a^
2,3,5-Trimethylpyrazine	0.735 ± 0.004 ^a^	0.577 ± 0.007 ^b^	0.467 ± 0.005 ^c^	0.314 ± 0.003 ^d^
2-Ethyl-6-methylpyrazine	0.408 ± 0.005 ^a^	0.419 ± 0.006 ^a^	0.263 ± 0.007 ^c^	0.295 ± 0.007 ^b^
2,5-Dimethylpyrazine	0.449 ± 0.003 ^b^	0.372 ± 0.007 ^c^	0.648 ± 0.015 ^a^	0.214 ± 0.006 ^d^
1,2-Dimethoxyethane	1.199 ± 0.016 ^c^	1.466 ± 0.022 ^b^	1.451 ± 0.011 ^b^	1.669 ± 0.038 ^a^
Allyl propyl sulfide	0.052 ± 0.002 ^c^	0.066 ± 0.001 ^b^	0.069 ± 0.002 ^b^	0.102 ± 0.001 ^a^
2-Pentylfuran	0.214 ± 0.005 ^b^	0.238 ± 0.007 ^a^	0.202 ± 0.001 ^b^	0.196 ± 0.001 ^b^
4,5-Dihydro-2-methyl-3(2 H) thiophenone	0.653 ± 0.003 ^c^	0.347 ± 0.002 ^d^	1.201 ± 0.008 ^b^	1.544 ± 0.009 ^a^
2-Phenyl-1,3-dioxolane-4-methanol D	0.037 ± 0 ^a^	0.032 ± 0.001 ^b^	0.023 ± 0.001 ^c^	0.024 ± 0.001 ^c^
2,2,4,6,6-Pentamethylheptane	0.179 ± 0.003 ^a^	0.186 ± 0.005 ^a^	0.145 ± 0.008 ^b^	0.13 ± 0.002 ^b^
2,4-Dimethylheptane	0.138 ± 0.002 ^a^	0.098 ± 0.001 ^c^	0.122 ± 0.002 ^b^	0.099 ± 0.002 ^c^
1-Heptene	0.127 ± 0 ^d^	0.258 ± 0.003 ^a^	0.213 ± 0.001 ^c^	0.246 ± 0.003 ^b^
3-Methyl valeric acid	0.249 ± 0.002 ^a^	0.244 ± 0.001 ^a^	0.246 ± 0.004 ^a^	0.249 ± 0.006 ^a^
Acetic acid	0.895 ± 0.01 ^c^	1.417 ± 0.009 ^a^	1.179 ± 0.034 ^b^	1.426 ± 0.007 ^a^
3-Methylbutanoic acid	0.174 ± 0.002 ^a^	0.129 ± 0.001 ^b^	0.135 ± 0.001 ^b^	0.135 ± 0.005 ^b^

Note: a–d: Mean ± SE (n = 3). Different superscript letters indicate significant differences (*p* < 0.05).

**Table 3:** WG Samples information.

Sampling Site	Sample Code	Age Group (Years)	Geographic Location	Altitude (m)	Vegetation Type	Slope Aspect	Sampling Date
S1	gs4-1	10	41°15′24″ N, 125°19′32″ E	485	Coniferous and broad-leaved mixed forest	Northeast	26 August 2025
	gs10-1	15					
	gs11-1	20					
	gs15-1	25					
S2	gs4-2	10	41°23′23″ N, 125°25′44″ E	443			
	gs10-2	15					
	gs11-2	20					
	gs15-2	25					
S3	gs4-3	10	41°30′11″ N, 124°57′19″ E	475			
	gs10-3	15					
	gs11-3	20					
	gs15-3	25					

## Data Availability

Data will be made available on request.

## Supplementary Materials

The following supporting information can be downloaded at: https://www.mdpi.com/article/10.3390/molecules31132315/s1, Table S1: WG Samples information.
