# Supplementary material for: Divergent Evolution of Volatile Compounds in Wild Ginseng Across Growth Years: Terpene Accumulation and Overall Pyrazine Decline Revealed by HS-GC-IMS
Source: Molecules. 2026 Jul 1;31(13):2315. doi: 10.3390/molecules31132315 (PMC13363443; doi:10.3390/molecules31132315)
Supplement: Supplementary file 1 [file molecules-31-02315-s001.zip › molecules-4345613-supplementary.pdf]

Compounds	gs4-1	gs4-2	gs4-3	gs10-1	gs10-2	gs10-3	gs11-1	gs11-2	gs11-3	gs15-1	gs15-2	gs15-3
Nerolidol	1.558	1.521	1.574	1.767	1.762	1.761	2.715	2.657	2.713	3.161	3.213	3.213
(E,E)-α-Farnesene	1.518	1.54	1.557	1.728	1.705	1.772	2.435	2.55	2.501	2.68	2.721	2.656
d-Longifolene	2.932	2.96	2.877	2.766	2.803	2.85	4.632	4.644	4.651	5.294	5.252	5.263
Isolongifolene	1.592	1.643	1.58	1.585	1.573	1.539	2.97	2.821	2.822	3.181	3.285	3.296
(+)-Limonene D	0.135	0.134	0.133	0.111	0.104	0.110	0.170	0.174	0.171	0.259	0.253	0.262
Camphene	0.584	0.614	0.633	0.544	0.538	0.539	1.213	1.193	1.236	2.025	2.011	2.167
(R)-α-pinene	0.827	0.840	0.857	0.575	0.580	0.579	1.041	1.016	1.110	1.626	1.609	1.744
β-Pinene P	1.034	1.033	1.013	0.622	0.608	0.626	1.012	1.094	1.118	1.584	1.569	1.606
β-ionone	1.494	1.521	1.508	2.099	2.105	2.039	2.416	2.424	2.409	2.875	2.898	2.995
(-)-Carvone	0.694	0.719	0.684	0.77	0.752	0.779	1.575	1.621	1.645	2.537	2.551	2.557
3-Octanone	0.251	0.269	0.253	0.127	0.126	0.116	0.529	0.512	0.600	0.843	0.838	0.867
3-Heptanone D	0.800	0.754	0.728	0.646	0.628	0.668	0.635	0.718	0.676	0.773	0.783	0.799
2-Hexanone	0.124	0.125	0.129	0.058	0.062	0.06	0.106	0.113	0.117	0.115	0.122	0.123
2-Butanone 3-hydroxy D	5.111	5.328	5.202	1.729	1.702	1.731	2.187	2.161	2.239	3.294	3.227	3.181
2-Pentanone	0.236	0.241	0.24	0.225	0.217	0.23	0.244	0.252	0.264	0.217	0.212	0.219
2,3-Pentanedione	0.339	0.343	0.35	0.361	0.351	0.347	0.304	0.309	0.3	0.394	0.399	0.394
2,3-Butandione	0.413	0.432	0.426	0.579	0.592	0.581	0.474	0.467	0.485	0.501	0.532	0.541
2-Butanone D	2.298	2.151	2.3	2.064	2.208	2.092	1.955	1.868	1.97	1.606	1.664	1.822
3-Pentanone	0.093	0.075	0.077	0.127	0.129	0.131	0.105	0.103	0.11	0.116	0.117	0.115
2-Pentanone, 3-methyl	0.015	0.015	0.013	0.015	0.015	0.015	0.014	0.017	0.014	0.022	0.023	0.023
4-methyl-3-Penten-2-one	1.053	1.058	1.016	0.816	0.801	0.797	0.732	0.705	0.696	0.744	0.719	0.729
5-Methyl-3-heptanone	2.857	2.896	2.785	2.737	2.658	2.689	2.774	2.688	2.759	2.966	2.988	2.898
1 -Hydroxy-2-propanone	0.397	0.418	0.4	0.642	0.642	0.629	0.502	0.486	0.485	0.61	0.605	0.592
(E)-2-Octenal	0.201	0.199	0.192	0.324	0.344	0.324	0.231	0.220	0.230	0.228	0.226	0.234
(E)-Hept-2-enal	0.318	0.332	0.306	0.461	0.474	0.452	0.362	0.338	0.353	0.307	0.303	0.318
(E)-2-Hexenal D	1.095	1.124	1.119	1.314	1.341	1.357	1.322	1.305	1.337	1.370	1.387	1.423
3-Methyl-2-butenal-D	1.567	1.641	1.587	1.485	1.466	1.463	1.211	1.145	1.218	1.317	1.274	1.327
Trans-2-pentenal D	0.18	0.187	0.177	0.154	0.159	0.158	0.159	0.155	0.155	0.174	0.168	0.177
Heptanal D	3.943	4.028	3.980	4.012	4.402	3.967	5.517	5.278	5.624	3.010	3.008	3.156
Hexanal D	7.279	7.316	7.429	9.254	9.4	9.281	7.97	7.897	7.974	8.087	8.083	8.127
Butanal	0.847	0.866	0.865	0.925	0.929	0.906	0.707	0.697	0.8	0.93	0.935	0.955
Propanal	1.678	1.718	1.781	2.774	2.6652	2.671	2.348	2.312	2.26	3.805	3.917	3.889
n-pentanal D	1.727	1.704	1.737	2.562	2.577	2.531	1.575	1.55	1.587	1.738	1.747	1.771
2-Methyl propanal	0.113	0.129	0.112	0.122	0.127	0.137	0.105	0.093	0.112	0.101	0.119	0.116
2-Phenylacetaldehyde	0.122	0.134	0.127	0.202	0.204	0.209	0.11	0.104	0.115	0.125	0.121	0.13

Compounds	gs4-1	gs4-2	gs4-3	gs10-1	gs10-2	gs10-3	gs11-1	gs11-2	gs11-3	gs15-1	gs15-2	gs15-3
Carveol	0.229	0.239	0.217	0.277	0.278	0.295	0.457	0.485	0.456	0.295	0.301	0.293
Linalool	0.372	0.384	0.364	0.418	0.411	0.416	0.528	0.536	0.561	0.553	0.56	0.570
1-Hexanol D	0.883	0.870	0.873	0.940	0.922	0.945	1.203	1.223	1.278	1.299	1.295	1.330
1-Pentanol D	0.175	0.185	0.176	0.346	0.341	0.352	0.188	0.184	0.19	0.191	0.183	0.196
3-Methyl butanol D	0.032	0.032	0.033	0.033	0.03	0.029	0.089	0.093	0.102	0.153	0.158	0.164
1-Butanol D	5.665	5.506	5.702	4.989	5.025	5.06	4.44	4.425	4.598	6.034	6.058	6.102
1-Propanol, 2-methyl	0.146	0.14	0.147	0.13	0.127	0.13	0.176	0.183	0.184	0.194	0.186	0.189
1-Propanol	13.614	13.212	13.706	14.048	14.343	14.016	12.601	12.483	12.778	12.701	12.768	12.759
1-Penten-3-ol	0.392	0.39	0.401	0.353	0.359	0.361	0.412	0.413	0.421	0.371	0.374	0.375
Acetic acid, hexyl ester	2.757	2.747	2.852	2.9	2.917	2.897	2.727	2.644	2.676	2.978	2.993	3.040
Ac. acetic ethyl ester D	1.065	1.165	1.133	2.322	2.214	2.107	1.437	1.408	1.624	2.85	2.826	2.939
n-Propyl acetate	0.082	0.069	0.084	0.168	0.175	0.172	0.16	0.149	0.154	0.098	0.104	0.113
Hexanoic acid methyl ester	1.046	1.061	1.094	1.259	1.286	1.267	1.287	1.263	1.252	1.233	1.243	1.217
Isobutyl propanoate	0.115	0.116	0.11	0.149	0.154	0.147	0.149	0.148	0.155	0.148	0.148	0.154
Methyl 2-methyl butyrate	1.07	1.075	1.089	0.987	0.975	1.021	1.43	1.427	1.444	1.227	1.19	1.224
Ethyl 2-methyl butanoate	0.165	0.170	0.170	0.187	0.189	0.188	0.310	0.316	0.304	0.294	0.286	0.302
n-Butyl lactate	0.182	0.168	0.170	0.165	0.150	0.159	0.159	0.163	0.157	0.158	0.150	0.146
(Z)-3-Hexenyl acetate	3.120	3.219	3.092	2.573	2.575	2.481	1.727	1.658	1.692	1.801	1.793	1.912
2-Furanmethanol acetate	0.039	0.046	0.042	0.047	0.049	0.054	0.046	0.041	0.043	0.042	0.041	0.045
2, 3, 5-Trimethylpyrazine	0.728	0.739	0.739	0.581	0.586	0.563	0.464	0.461	0.477	0.310	0.314	0.319
2-Ethyl-6-methylpyrazine	0.398	0.413	0.413	0.421	0.427	0.408	0.267	0.250	0.272	0.286	0.290	0.308
2,5-Dimethylpyrazine	0.447	0.446	0.454	0.369	0.386	0.361	0.666	0.619	0.6588	0.2088	0.208	0.225
1,2-Dimethoxyethane	1.179	1.188	1.23	1.455	1.509	1.435	1.449	1.433	1.472	1.637	1.624	1.745
Allyl propyl sulfide	0.055	0.053	0.049	0.065	0.065	0.067	0.066	0.068	0.073	0.01	0.101	0.104
2-Pentyl furan	0.215	0.205	0.223	0.242	0.224	0.249	0.202	0.201	0.203	0.198	0.196	0.195
4,5-Dihydro-2-methyl-3(2 H)thiophenone	0.647	0.655	0.657	0.344	0.352	0.346	1.203	1.186	1.215	1.531	1.538	1.562
2-Phenyl-1.3-dioxolane-4-methanol-D	0.037	0.038	0.037	0.03	0.031	0.034	0.023	0.0211	0.024	0.023	0.025	0.024
2,2,4,6,6-Pentamethylheptane	0.179	0.175	0.184	0.176	0.195	0.186	0.132	0.144	0.158	0.129	0.126	0.134
2,4-Dimethylheptane	0.134	0.141	0.138	0.099	0.096	0.099	0.12	0.12	0.126	0.097	0.097	0.102
3-Methyl valeric acid	0.252	0.246	0.250	0.245	0.242	0.244	0.239	0.246	0.253	0.254	0.237	0.255
1-Heptene	0.127	0.127	0.127	0.258	0.263	0.254	0.215	0.212	0.211	0.246	0.251	0.242
Acetic acid	0.876	0.909	0.899	1.405	1.411	1.435	1.223	1.202	1.112	1.436	1.431	1.412
3-Methy lbutanoic acid	0.175	0.176	0.171	0.131	0.129	0.127	0.134	0.135	0.136	0.145	0.128	0.133
